# Controllable Electrochromic Polyamide Film and Device Produced by Facile Ultrasonic Spray-coating

**DOI:** 10.1038/s41598-017-11862-1

**Published:** 2017-09-20

**Authors:** Huan-Shen Liu, Wei-Chieh Chang, Chin-Yen Chou, Bo-Cheng Pan, Yi-Shan Chou, Guey-Sheng Liou, Cheng-Liang Liu

**Affiliations:** 1 Institute of Polymer Science and Engineering, National Taiwan University, Taipei, 10617 Taiwan; 2 Department of Chemical and Materials Engineering, National Central University, Taoyuan, 32001 Taiwan

## Abstract

Thermally stable TPA-OMe polyamide films with high transmittance modulation in response to applied potential are formed by facile ultrasonic spray-coating. Four processing conditions (Film A, Film B, Film C and Film D) through tuning both solution concentrations and deposition temperatures can be utilized for the formation of wet and dry deposited films with two film thickness intervals. The electrochromic results show that the dry deposited rough films at higher deposition temperature generally reveal a faster electrochromic response, lower charge requirements (Q) and less conspicuous color changes (smaller optical density change (ΔOD) and lightness change (ΔL*)) during the oxidation process as compared to the wet deposited smooth films at lower deposition temperature. Moreover, thicker electrochromic films from increased solution concentration exhibit more obvious changes between coloration and bleaching transition. All these four polyamide films display colorless-to-turquoise electrochromic switching with good redox stability. The large scale patterned electrochromic film and its application for assembled device (10 × 10 cm^2^ in size) are also produced and reversibly operated for color changes. These represent a major solution-processing technique produced by ultrasonic spray-coating method towards scalable and cost-effective production, allowing more freedoms to facilitate the designed electrochromic devices as required.

## Introduction

The phenomenon of electrochromism is macroscopically explained by properties of the change and evocation/bleaching of color as a consequence of the redox reaction observing in the electrochromic materials, and resulted from the generation of different electronic absorption band on switching between a transmissive (bleaching) state and an absorptive colored one, or between two absorptive colored states with different hues^1–11^. Numerous electrochromic applications include their use in protective sunglass, smart window, anti-glare mirrors, light-reflective/light-transmissive display panel and adaptive camouflage for the military assets, etc. From materials points of view, many electrochromic organic-based monomeric^12^ or polymeric^1–8^ molecules can even outperform their inorganic counterparts in some aspects due to their advantageous features such as relatively structural design flexibility for color-tuning, easy-processing and potentially low-cost. Especially, conjugated conducting polymers^2–5^, metallopolymers^13–16^ and arylamine-based polymers^7^ are main types of polymeric electrochromic materials, widely investigated for candidates of electrochromic devices (ECDs) in recent years.

In order to fully exploit the potential of electroactive device, it is necessary to achieve simple film-forming (especially in non-vacuum apparatus) and low manufacturing cost production by use of a wide varieties of solution-processable electrochromic materials. One emerging technique capable of satisfying these requirements is spray deposition. Previously, spray technology has already been successfully utilized for the deposition of electrochromic conjugated polymers^17–29^ and metal oxide^30–32^ film. Ultrasonic spraying is one of typically used types in which formation of standing waves in the precursor solution through ultrasonic atomization process allows to be delivered onto the underlying substrate with a controlled gas flow. Therefore, ultrasonic spray-coating technique is highly adaptable to high throughput and large volume fabrication with advantages of good reproducibility and precise coating if spraying ink solution consisting of good material solubility, volatile solvent used and suitable viscosity is readily prepared. Our group has been synthesizing and characterizing a series of solution-processable polyamides aiming at their anodically electrochromic application^33–40^, and most of these films were processed by traditional spin-coating method. Solution-processable electrochromic polymers which can be dissolved in common organic solvents and spray-coated onto substrate need to be further exploited in order to possess long cyclic life, bleaching/coloring efficiency and apply for large area and patterned solid state devices. Therefore, how to control the processing parameters during the ultrasonic spray-coating process plays an important role for deposition of polyamides films with good electrochromic behavior.

**Figure 1:**
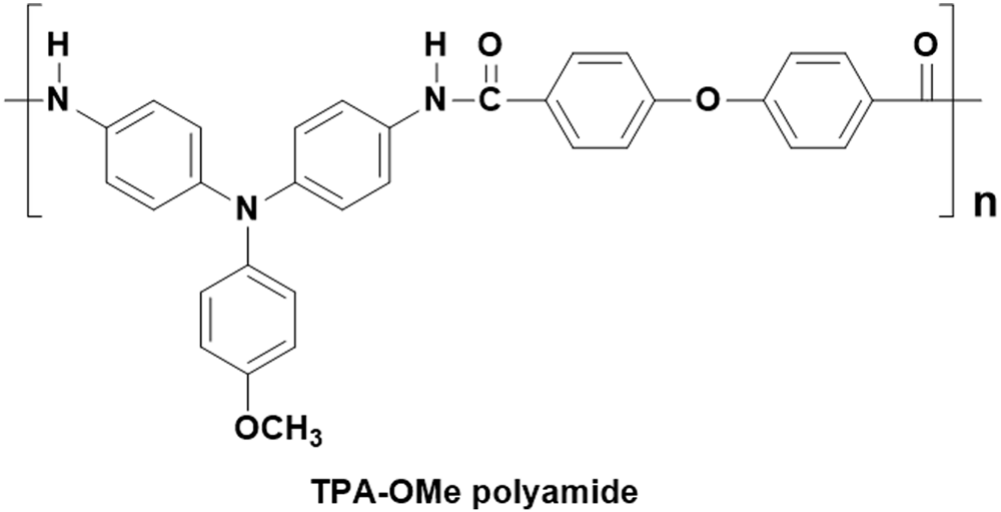
Chemical structures of electrochromic TPA-OMe polyamide.

Here, we report a detailed investigation of the influence of processing parameters on the quality and electrochromic properties of the resulting ultrasonic spray-coated methoxy-substituted triphenylamine-containing polyamides (TPA-OMe) films (chemical structure in Fig. 1). Solution-processable polyamide with high thermal stability and initial optical transparency is believed to possess good properties for electrochromic use. A spray nozzle with programmable movement is used for spraying onto ITO substrates placed on a stationary heating plate. Selected processing parameters, polymer solution concentrations and deposition temperatures, were investigated for factorial design of electrochromic testing experiments. Wet/dry deposited electrochromic polyamides film (controlled by heating temperature) as well as film thickness (controlled by ink formulation) command the eventual performance metrics such as response time, optical density change (ΔOD), color efficiency (*η*), cycling stability capable of switching between the bleaching (neutral) and coloring (oxidized) state. A reliable and reproducible large scale spray deposition process demonstrated here are suitable for utilizing as integration into solid-state patterned ECD with the application of appropriate electrical potentials.

## Results and Discussion

**Figure 2:**
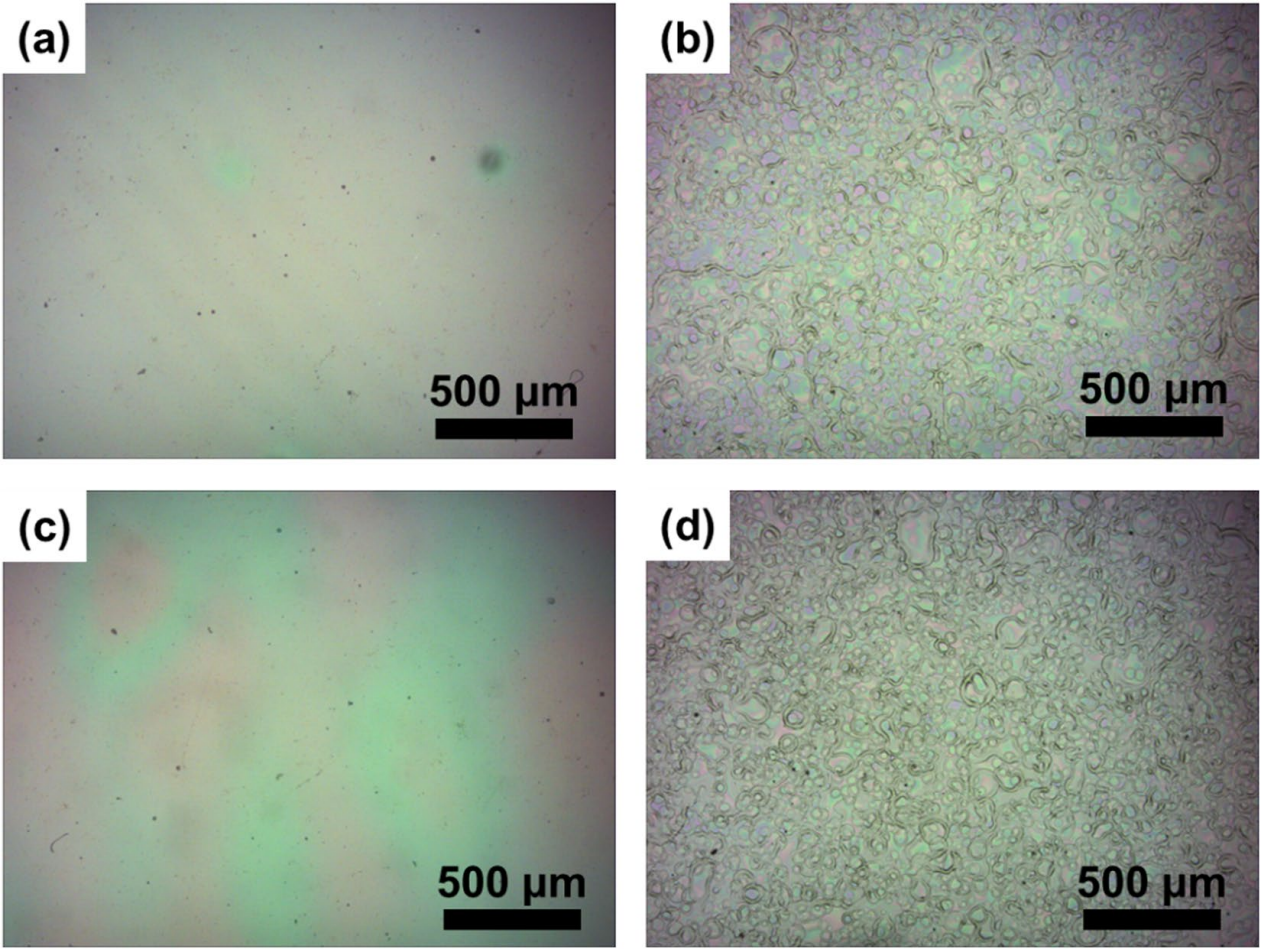
Optical micrographs of ultrasonic spray-coated (**a**) **Film A**, (**b**) **Film B**, (**b**) **Film C** and (**d**) **Film B**.

The ultrasonic spray process used in this study were proven to be facile and versatile to fabricate the polymer thin film and widely applied because the spray process can be done at high throughput and compatible with large scale fabrication. A preliminary investigation was performed to find the optimized parameters to deposit layers with the desired physical properties in terms of thin film thickness, coverage and morphology. Fig. S1 shows the schemes of the computer numeric controlled spray system equipped with a servo-motorized nozzle head. Through the variations in two controllable parameters, polymer solution concentrations and substrate heating temperatures, four electrochromic films in different processing conditions can be investigated. **Film A** is obtained from TPA-OMe polyamide solution of 8 mg ml^−1^ and heated at 40 °C; **Film B**: 8 mg ml^−1^ and 80 °C; **Film C**: 16 mg ml^−1^ and 40 °C; **Film D**: 16 mg ml^−1^ and 80 °C. It should be noted that four processing conditions for ultrasonic spray-coated electrochromic films were used here since the significant changes in electrochromic properties between all these films can be systematically observed and compared. Examples of optical images of coated TPA-OMe thin film under different deposition conditions are displayed in the Fig. 2. The impact of thermal annealing supplied during the film formation process clearly indicates the change in the morphologies of prepared **Film A** (Fig. 2a), **Film B** (Fig. 2b), **Film C** (Fig. 2c) and **Film D** (Fig. 2d)). TPA-OMe polyamide thin film prepared at lower (higher) heating temperature results in wet (dry) deposition, caused by drying rate of polymer solution that is lower (higher) than deposition rate. Therefore, it is found that the surface of polymer thin film is filled with interconnected and smooth layer at deposition temperature of 40 °C (**Film A** and **Film C**) and spherical ball-like structure with high porosity feature observed in thin film prepared at 80 °C (**Film B** and **Film D**). These results also show no significant morphological change by concentration variation. Fig. S2 shows that profilometry measurements of the surface roughness of TPA-OMe polyamide thin films as the function of concentrations of polyamide ink and heating temperatures during the spray process, and Table 1 summarizes the average thickness for these four films. The average film thickness is positively correlated with the concentration of polymer solution (~300 and ~600 nm for solution of 8 and 16 mg ml^−1^, respectively). However, it can be seen that the maximum peak-to-valley height at a fixed length scale of 0.6 μm is relatively small for wet deposited film (**Film A** and **Film C**) as compared with dry deposited film (**Film B** and **Film D**), and thus the wet deposited film is quite flat with a small variation in thickness of ± 11 and ± 28 nm for **Film A** and **Film C**, respectively. The roughness and inhomogeneity increase significantly with the annealing temperature but slightly with the polymer concentration. Especially, individual spray droplets dry independently and feed solution is partially overlapped and not well-accumulated under the dry deposited condition. In these primary analytical results of ultrasonic spray-coated electrochromic polymer thin films, the solvent evaporation kinetic is mainly altered by manipulating the ink formation and deposition temperature and important to further obtain the active electrochromic layer with required morphologies.

**Table 1:** Optical and electrochemical data collected for coloration efficiency measurements of ultrasonic spray-coated TPA-OMe polyamide films under different processing conditions.

Film	Thickness (nm)	Potential (V)	Response time (s)		Cycling times	∆OD	*Q* (mC cm^−2^)	*η* (cm^2^ C^−1^)	Decay in *η* (%)
			Coloring	Bleaching					
**Film A**	283 ± 11	1.0	4.5	2.4	1	1.10	3.41	323	-
					100	1.07	3.40	315	2.48
**Film B**	280 ± 186	0.9	4.2	2.2	1	0.73	1.92	380	-
					100	0.72	1.90	379	0.26
**Film C**	583 ± 28	1.1	5.1	2.8	1	2.14	5.43	394	—
					100	2.09	5.35	390	1.01
**Film D**	645 ± 244	1.0	3.4	1.8	1	1.22	3.57	342	—
					100	1.18	3.51	336	1.75

**Figure 3:**
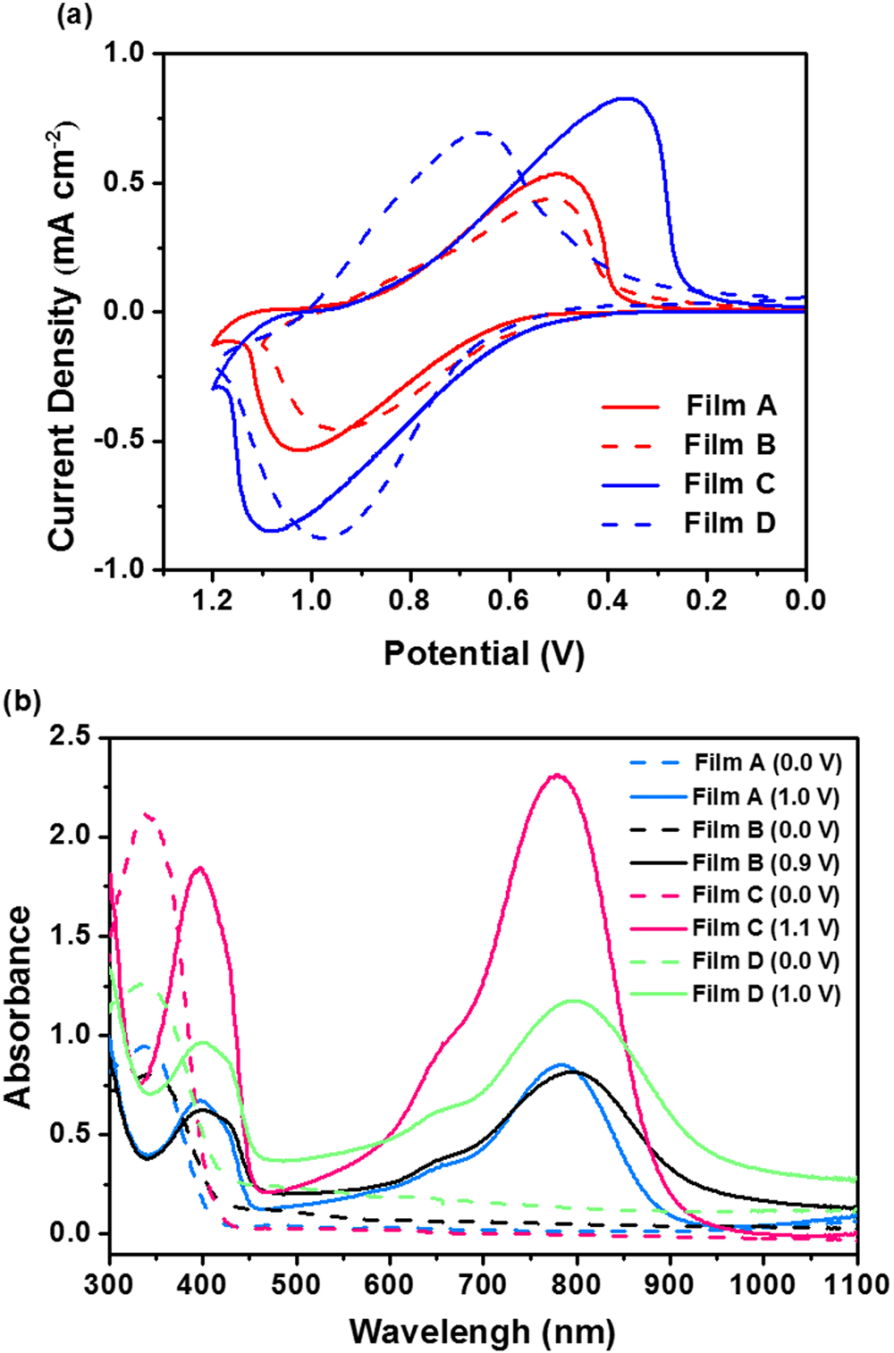
(**a**) Single scan cyclic voltammetry and (**b**) spectroelectrochemistry of four ultrasonic spray-coated TPA-OMe polyamide films on ITO glass substrate (area: 1 × 2 cm^2^) under different processing conditions.

In order to compare the electrochemical properties of four thin films with different processing conditions, the cyclic voltammetric studies for oxidation/reduction of TPA-OMe polyamide films were performed. Fig. 3a shows the stable cyclic voltammograms of **Film A**, **Film B**, **Film C** and **Film D**, respectively, coated on a 1 × 2 cm^2^ ITO glass at a scan rate of 50 mV s^−1^. Each films have one oxidation peak (in a potential range between 0.95 and 1.13 V) during the anodic scan and one reduction peak (in a potential range between 0.38 and 0.66 V) during cathodic scan, which is in consistent with previous redox processes reported for the similar polyamides by spin-coating^40^. It is noted that controlled solution concentrations and annealing temperatures greatly affect the current density and redox couple of the polymer film. First, quasi-reversible electrochemical behaviors were shown in four films, as evidenced by almost equal anodic and cathodic peak current density. However, current density passing through the coated film increases gradually with the increase of spray ink concentration (**Film A** vs **Film C** (solid line); **Film B** vs **Film D** (dash line)) regardless of the annealing temperatures, most probably since the higher material concentration/coverage on the substrate leads to higher electrochemical activity. The potential separation between the oxidation and reduction couple is 0.50, 0.44, 0.69 and 0.30 V, for **Film A**, **Film B**, **Film C** and **Film D**, respectively, indicating that the voltage difference between redox waves decreases with an increase of annealing temperature (at a fixed polymer concentration). This result implies that rapid color change can be expected for the dry deposited film as a possible result of its faster charge transfer behavior across the rough surface. Fig. 3b shows the spectroelectrogram of four films in bleached and colored state under an applied potential of 0 V and 0.9~1.1 V, respectively, revealing the anodically colored polymeric electrochromic films. Initially, a well-defined absorption band centers at ~340 nm, which can be attributed to neutral state of TPA-OMe polyamide film. With further applying the potential bias, the evolution of new absorption bands could be observed at ~398 and ~787 nm. This typically represents polaronic absorption bands with cationic radical delocalized within triphenylamine (TPA) units along the polyamide backbone^40^. Here, absorbance intensity in specific wavelength region varies with the processing conditions. The absorbance in the main characteristic bands increases with the concentration, judging from the UV absorption at ~339 nm before applying potential and visible/near-infrared absorption at ~398 nm/~787 nm after applying potential. It suggests that the oxidation of TPA moieties in the TPA-OMe polyamide performs more significantly based on the thicker polymer film (**Film C** and **Film D**). Especially, **Film C** exhibits such high absorption after oxidation process mainly because of small thickness variation from thick film and higher applied potential to reach color change. Besides, an attenuation in the visible absorbance as well as an increase in absorption band edge (wavelength of more than 900 nm in near-infrared region) with increasing the annealing temperature from 40 °C to 80 °C can be determined, which is primarily due to scattering effect of rough polymer surface from dry deposited film.

**Figure 4:**
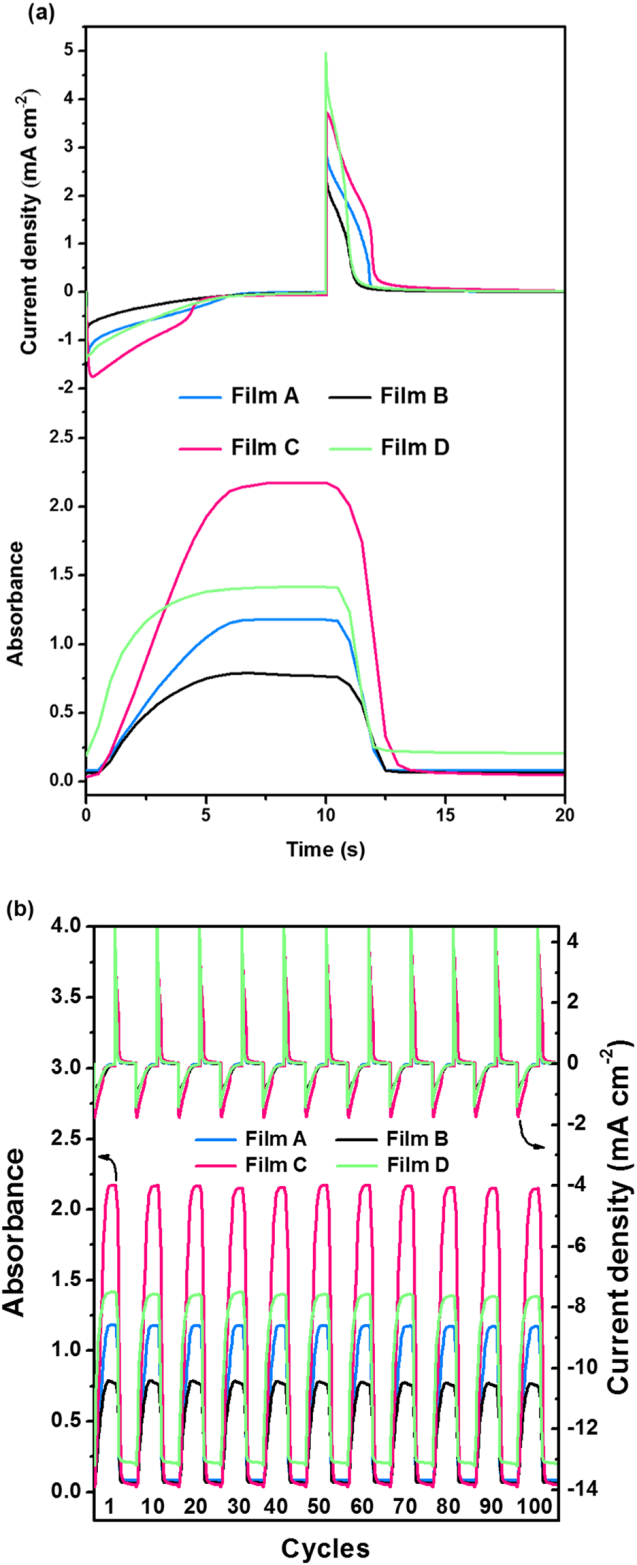
Chronoamperometric and chronoabsorptometric (at 787 nm) experiments for four ultrasonic spray-coated TPA-OMe polyamide films (area: 1 × 2 cm^2^) by applying a potential step and cycle time of 20 s obtained (**a**) at the first cycle and (**b**) during 100 cycles.

The electrochromic switching time and stability are important for evaluation of coloring/breaching kinetics. The switching time is the time required to the electrochromic film to change from one redox state to the other and vice versa, defined as the time required for reaching 90% of formation of final color after switching the potential here. Under a stepping potential input of 0.9 ~ 1.1 V or 0 V with a regular interval of ~5 s at 787 nm, the current density and optical response in the first cycle were illustrated in Fig. 4a. The electrical output of four thin films shown in upper Fig. 4a presents an anodic peak current (negative current value) and a cathodic peak current (positive current value) for the color state and bleached state, respectively. Chronoabsorptometric measurement can be performed to determine the optical absorbance changes, as shown in the lower Fig. 4a. Table 1 summarizes the optical and electrochromic parameters, such as switching time, ΔOD, Q, and *η*, determined from four ultrasonic spray-coated films in electrolyte solution. The electrochromic TPA-OMe polyamide film shows a decrease for both anodic and cathodic peak current density as the annealing temperature is enhanced (upper Fig. 4a), which proves a lower activated current to be coloring/bleaching for the dry deposited film with rough surface upon switching between the neutral and oxidized state. For **Film A**, the switching time for coloring and bleaching is 4.5 and 2.4 s, respectively, which is comparable to the results of spin-coated TPA-OMe thin film given in the literature^40^. The oxidation (coloring) proceeds more slowly than reduction (bleaching), suggesting typical p-type doping of electrochromic polymer^40^. However, the switching time observed in rougher **Film B** (**Film D**) surface fabricated at higher annealing temperature is much shorter than the smooth **Film A** (**Film C)** to reach the same absorbance modulation. Among all, data from a rather thick and smooth film by increasing the polymer solution (**Film C**) demonstrate a longest switching time. The ΔOD increases linearly with the film thickness controlled by polymer solution concentration. These results indicate that the ΔOD of thin film from 16 mg ml^−1^ is almost two times than that from 8 mg ml^−1^, ΔOD of 2.14 (1.22) for **Film C** (**Film D**) and of 1.10 (0.73) for **Film A** (**Film B**), respectively. The obtained smaller ΔOD for color transition of thin films annealed at higher temperature is also in consistent with the decrease of the absorption intensity of band at wavelength of 787~798 nm for dry deposited films (**Film B** and **Film D**). To quantify the electrochromic performance, the *η* of films at four different processing conditions, defined by proportionality factor between ΔOD and Q, is also evaluated. From the upper Fig. 4a, the charge amounts though the electrochromic layers can be estimated when switching between 0 and 0.9 ~ 1.1 V (see Table 1). The Q value of **Film A** amounts to 3.41 mC cm^−2^ when it is colored/bleached. In comparison with **Film A**, the Q value of **Film B** decreases for coloring/bleaching transition (1.92 mC cm^−2^), mainly due to the lower charge requirement from rough surface of the dry deposited **Film B**. Similar trend in Q value can be judged for coated thin films from higher concentration (**Film C** and **Film D**). The *η* value of **Film A** obtained for a certain amount of charge transport across the film as a function of ΔOD is calculated to be 323 cm^2^ C^−1^, which is fairly good compared to that of the spin-coated film in our previous reported electrochromic polyamides^40^. For electrochromic behavior at higher annealing temperature (**Film B**; still at a solution concentration of 8 mg ml^−1^), the drying time is longer than the time interval between the two subsequent droplets and all the droplets combine to form a rough surface, suggesting that the charges can easily be transferred across the polyamide film. Therefore, it can be expected that a higher *η* value in according with the small decrease in ΔOD at 787 nm and more significant decrease in Q value is obtained thanks to the rough surface which is generally considered to be advantageous for the higher degree of ion diffusion across the dry deposited film in the electrolytic solution. However, the reverse trend in *η* value between **Film C** and **Film D** (from higher concentration of 16 mg ml^−1^) since the slightly Q value decreasing can not compensate for the reduced ΔOD of dry deposited film as the annealing temperature increases. The strong couples between the electrochromic kinetic studies with these parameters discussed above demonstrate that the dry deposited TPA-OMe polyamide film at a higher deposition temperature generally reveals the faster electrochromic response and lower charge requirements but less conspicuous color changes during the oxidation process as compared to the wet deposited film. Electrochemical stability and reversibility for the long term switching between the coloring and bleaching states are important characteristics for electrochromism. Ultrasonic spray-coated films were also subjected to extended cycling tests and the obtained result is presented in Fig. 4b. All these four films possess good electrochromic stability under atmospheric condition, less than 2.48% decay in *η*, after the completion of 100 cycles.

**Figure 5:**
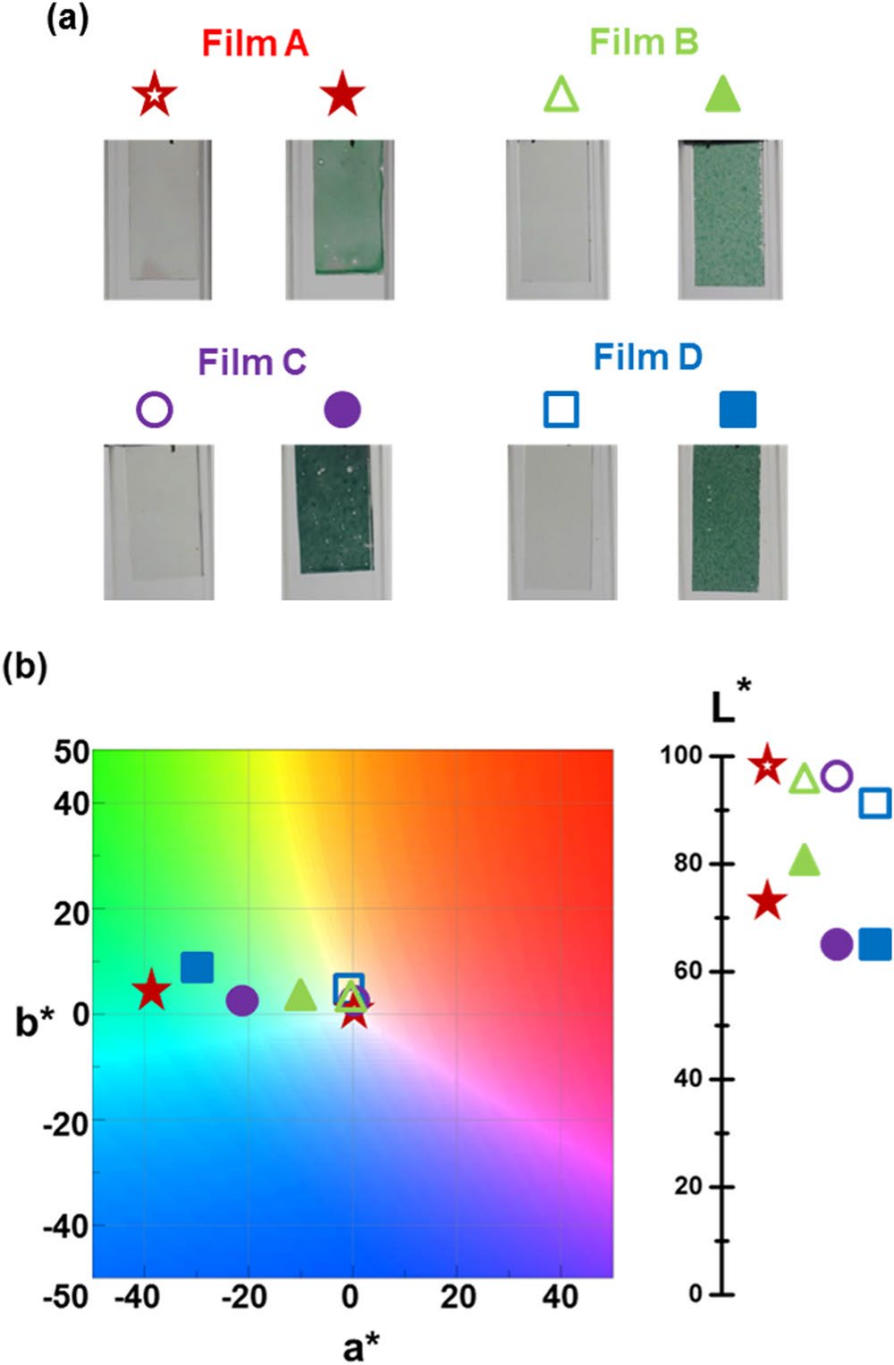
(**a**) Photograph and (**b**) corresponding CIE 1976 color coordinates of four ultrasonic spray-coated TPA-OMe polyamide films (area: 1 × 2 cm^2^) in the neutral and oxidized state.

The photographs of color change between electrochemically produced states based on four electrochromic films are shown in Fig. 5a. A noticeable and visible difference for coloring/beaching state can be detected. Meanwhile, in order to evaluate color changes of TPA-OMe polyamide film with respect to the human eye, the calculated (L*, a*, b*) coordinates, uniform color space defined by the Commission Internationale de I’Eclairage (CIE) in 1976, are also summarized at each applied potential in Fig. 5b and Table 2. L* is the lightness variable of the sample, whereas a* and b* correspond to the two antagonistic chromatic processes (red/green and yellow/blue). In the neutral state, TPA-OMe polyamide films exhibit L* from 97.8 (97.3) for the wet deposited film to 96.4 (91.9) for dry deposited film. In comparison to oxidized polymer film, L* varies from 74.3 (65.3) for the wet deposited film to 81.4 (64.6) for dry deposited film. Concurrently, the lightness change (ΔL*) varies from 23.5 (32.0) for the wet deposited film to 15.0 (27.3) for dry deposited film as the transparent TPA-OMe polyamide is colored through the intensely absorbing chromophore, indicating the ability to modulate the luminance based on film processing conditions. It should be noted here that the steady reduction in L* or enhancement in ΔL* for the wet deposited film (processing at lower deposition temperature) after oxidation process corresponds to the relatively darker color represented in Fig. 5a because of its smooth surface with higher ΔOD. Besides, the thicker TPA-OMe film also exhibits more significant ΔL*. This observation also agrees well with the visible absorption shown in Fig. 3b as large amount of visible light is absorbed by the thicker polymer film coated from higher concentration. Correspondingly, a* and b* color coordinates approach center zero, which presents the capacity of films to achieve a highly transparent state in the neutral state. The (L*, a*, b*) coordinates nicely exhibit the changes that take place for the transition of achromatic to turquoise. The a* coordinate moves to the negative direction; the b* coordinate moves to positive direction although the final value shows a low saturation.

**Table 2:** Colorimetry properties, according to the CIE (L*, a*, b*) chromaticity coordinates, of ultrasonic spray-coated TPA-OMe polyamide films under different processing conditions.

Film	Potential (V)	Color coordinate			ΔL*
		L*	a*	b*	
Film A	0.0	97.8	0.6	0.4	23.5
	1.0	74.3	−31.9	5.2	
Film B	0.0	96.4	−0.4	2.5	15.0
	0.9	81.4	−12.7	3.0	
Film C	0.0	97.3	1.3	1.4	32.0
	1.1	65.3	−22.0	0.2	
Film D	0.0	91.9	−1.0	5.5	27.3
	1.0	64.6	−29.5	7.7	

**Figure 6:**
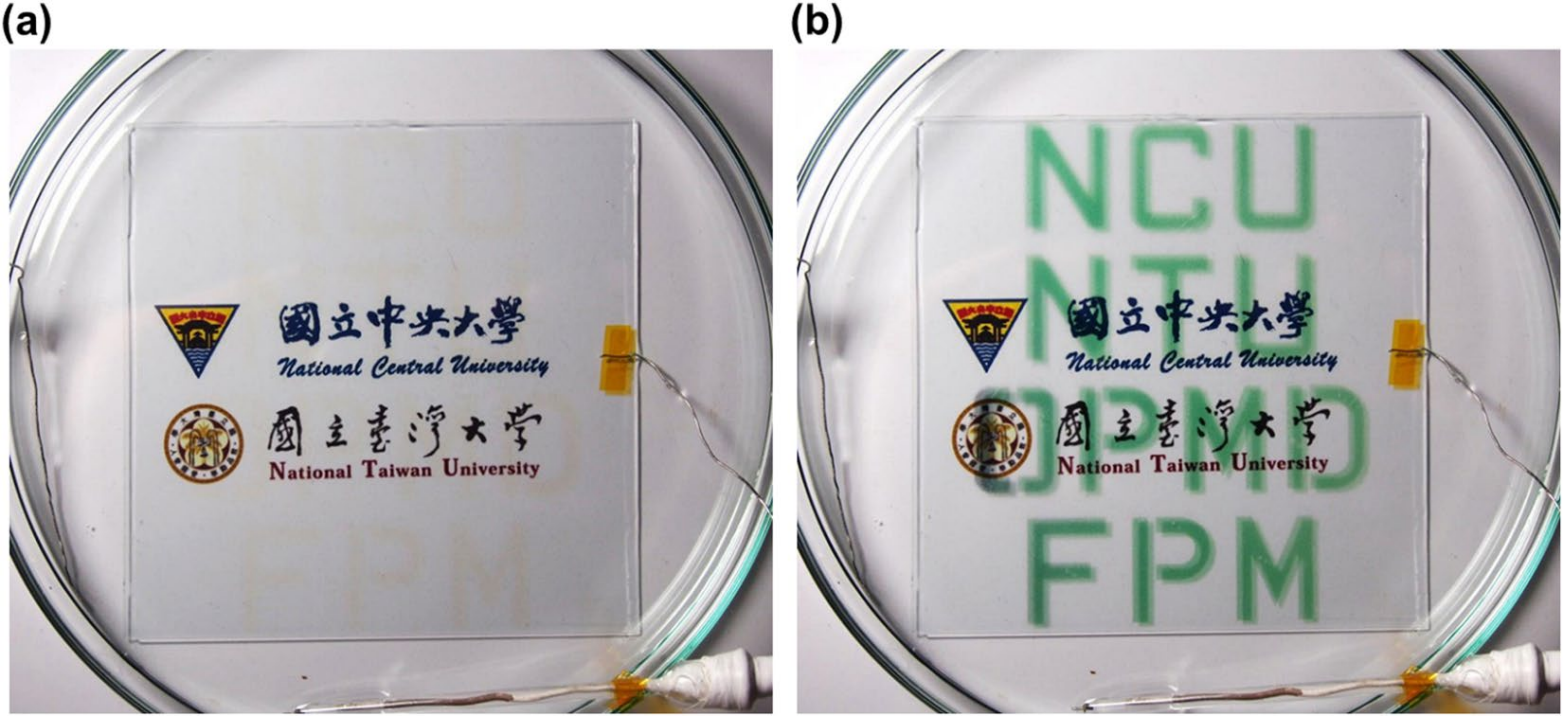
Photograph of patterned TPA-OMe polyamide film on 10 × 10 cm^2^ ITO glass in 0.1 M TBAP/acetonitrile solution in the (**a**) neutral and (**b**) oxidized state.

Since the ultrasonic spray-coating technique is easily controlled, the letters formed by dry deposited TPA-OMe film (same as **Film D** processing condition) can be prepared on 10 × 10 cm^2^ ITO glass by computer-aided ultrasonic spray-coating via a mask. The electrodes were immersed into the tetrabutylammonium perchlorate (TBAP)/acetonitrile electrolyte upon applying potential. Fig. 6 exhibits the color changing process of patterned letters between high optical transparency in the neutral state without applying potential (Fig. 6a) and turquoise in the oxidized state with applying the potential of 0.95 V for 1 min (Fig. 6b). An important result is that coloring/bleaching is stably achieved for ultrasonic spray-coated patterned film in a large area at least for several cycles. These patterned TPA-OMe polyamide films with a thickness of ~600 nm were also assembled for the application in ECD, as shown in Fig. S3. As indicated in the spectroelectrochemical studies of **Film D** presented in Fig. 3b, the visible absorption of TPA-OMe polyamide film colors extensively with applied potential. As a result, the letters in ECD reversibly bleaches and colors at 0 and 1.5 V, respectively, between the neutral and oxidized state. Fully functional electrochromic colorless-to-turquoise devices with reasonable ambient redox stabilities were demonstrated by ultrasonic spray-coated film. Therefore, different patterns and careful selection of electrochromic polymers with different color switching can be ultrasonic spray-coated onto varieties of substrates and designed for sophisticated templates in the near future.

## Conclusion

In summary, we have demonstrated the ultrasonic spray-coated polyamide electrochromic film, which can be used to study the variation of electrochromic layer through changing the ink solution concentrations and deposition temperatures. The ink formulation can be directly related to film thickness, whereas the heating temperature during coating process has a strong impact on the roughness of deposited film. The strong couples between the kinetic studies with these two processing parameters demonstrate that the dry deposited TPA-OMe polyamide film at higher deposition temperature reveals a faster electrochromic response, lower charge requirements and less conspicuous color changes (smaller ΔOD and ΔL*) during the oxidation process as compared to the wet deposited film at lower deposition temperature. On the other hand, manipulation in solution concentrations almost shows reverse trend in electrochromic behavior as compared to deposition temperature. All these four films with different coating conditions sustain duty cycles of neutralization/oxidation switching without color fatigue. The scalable ultrasonic spraying process developed here is easily amenable for large area and patterned film, and is well applied for ECD changing its transmittance from transparent to turquoise with good electrochromic performance.

## Methods

All the chemical reagents were purchased from commercial suppliers and used as received, unless stated otherwise. The electrochromic polyamide, TPA-OMe, was prepared according to our previously reported synthetic procedure (*M*_*W*_~43,000; PDI~1.34)^33^.

Optical micrographs were obtained by Leica 2700 M. Cyclic voltammograms were collected with a CHI 611B electrochemical analyzer and three-electrode cell, with a polymer film on ITO as working electrode, homemade Ag/Ag^+^ as non-aqueous reference electrode and a platinum wire as auxiliary electrode, in acetonitrile (CH_3_CN) containing 0.1 M tetrabutylammonium perchlorate (TBAP) as supporting electrolyte at room temperature. Spectroelectrochemical measurements were performed by coupling both the Agilent 8453 UV/Vis spectrophotometer and CHI potentiostat/galvanostat. The surface height and thickness of coated polymer films were measured using Microfigure Measuring Instrument SUFCORDER (ET3000, Kosaka Laboratory Ltd.). Colorimetry measurements were obtained under the potentiastatic control by use of a Jasco V-650 spectrophotometer. Photographs of electrochromic polymer film and devices were taken with Olympus digital camera.

Fabrication of electrochromic TPA-OMe polyamide film was realized by ultrasonic spray atomization and deposition. The polymer ink solution in dimethylacetamide (DMAc) is diluted to 8 and 16 mg ml^−1^, respectively, to judge the film thickness effect for electrochromism. The ultrasonic spray nozzle (Sono-Tek; 120 kHz) is mounted onto a custom-built X-Y-Z movable scanner and the polymer solution flow rate is controlled by the syringe pump, while nitrogen at a flow of 0.01 mPa was used as a carrier gas. Major spray parameters in order to be adjusted for obtaining desired spray characteristics are flow rate (8~10 ml hr^−1^), nozzle-to-substrate distance (approximately 4 cm) and X-Y moving speed of 0.5 cm s^−1^. The nozzle is moved in a pre-programmed zig-zag pattern along X and Y direction to spray an area of up to 10 × 10 cm^2^ with a heatable stage at 40 and 80 °C, respectively. Therefore, heating temperatures result in wet/dry deposition. The TPA-OMe polyamide film was also assembled for ECD. ITO glass electrode (10 × 10 cm^2^) was ultrasonic spray-coated with TPA-OMe polyamide using **Film ﻿D﻿** condition though the shadow mask. A gel electrolyte based on PMMA (1.25 g) and LiBF_4_ (0.15 g) was plasticized with propylene carbonate (2.75 g) to form a highly transparent and conductive gel. The gel electrolyte was spread on the polymer-coated side of the electrode, and another ITO glass electrode was covered onto top of electrolyte layer.

## Electronic supplementary material

Controllable Electrochromic Polyamide Film and Device Produced by Facile Ultrasonic Spray-coating

## References

[1] Mortimer RJ, Dyer AL, Reynolds JR (2006). Electrochromic Organic and Polymeric Materials for Display Applications. Displays.

[2] Beaujuge PM, Amb CM, Reynolds JR (2010). Spectral Engineering in pi-Conjugated Polymers with Intramolecular Donor-Acceptor Interactions. Acc. Chem. Res..

[3] Beaujuge PM, Reynolds JR (2010). Color Control in pi-Conjugated Organic Polymers for Use in Electrochromic Devices. Chem. Rev..

[4] Amb CM, Dyer AL, Reynolds JR (2011). Navigating the Color Palette of Solution-Processable Electrochromic Polymers. Chem. Mater..

[5] Neo WT, Ye Q, Chua SJ, Xu JW (2016). Conjugated Polymer-Based Electrochromics: Materials, Device Fabrication and Application Prospects. J. Mater. Chem. C.

[6] Thakur VK, Ding GQ, Ma J, Lee PS, Lu XH (2012). Hybrid Materials and Polymer Electrolytes for Electrochromic Device Applications. Adv. Mater..

[7] Yen HJ, Liou GS (2012). Solution-Processable Triarylamine-Based Electroactive High Performance Polymers for Anodically Electrochromic Applications. Polym. Chem.

[8] Kline WM, Lorenzini RG, Sotzing GA (2014). A Review of Organic Electrochromic Fabric Devices. Color. Technol..

[9] Gillaspie DT, Tenent RC, Dillon AC (2010). Metal-oxide Films for Electrochromic Applications: Present Technology and Future Directions. J. Mater. Chem..

[10] Granqvist CG (2014). Electrochromics for Smart Windows: Oxide-based Thin Films and Devices. Thin Solid Films.

[11] Cai GF, Wang JX, Lee PS (2016). Next-Generation Multifunctional Electrochromic Devices. Acc. Chem. Res..

[12] Navarathne D, Skene WG (2013). Towards Electrochromic Devices Having Visible Color Switching Using Electronic Push-Push and Push-Pull Cinnamaldehyde Derivatives. ACS Appl. Mater. Interfaces.

[13] Higuchi M (2009). Electrochromic Organic-Metallic Hybrid Polymers: Fundamentals and Device Applications. Polym. J..

[14] Hu CW, Sato T, Zhang J, Moriyama S, Higuchi M (2013). Multi-colour Electrochromic Properties of Fe/Ru-based Bimetallo-supramolecular Polymers. . J. Mater. Chem. C.

[15] Han FS, Higuchi M, Kurth DG (2008). Metallosupramolecular Polyelectrolytes Self-assembled from Various Pyridine Ring-substituted Bisterpyridines and Metal Ions: Photophysical, Electrochemical, and Electrochromic Properties. J. Am. Chem. Soc..

[16] Chen BH (2015). Printed Multicolor High-Contrast Electrochromic Devices. ACS Appl. Mater. Interfaces.

[17] Dyer AL, Thompson EJ, Reynolds JR (2011). Completing the Color Palette with Spray-Processable Polymer Electrochromics. ACS Appl. Mater. Interfaces.

[18] Beaujuge PM, Ellinger S, Reynolds JR (2008). Spray Processable Green to highly Transmissive Electrochromics via Chemically Polymerizable Donor-Acceptor Heterocyclic pentamers. Adv. Mater..

[19] Mortimer RJ, Graham KR, Grenier CRG, Reynolds JR (2009). Influence of the Film Thickness and Morphology on the Colorimetric Properties of Spray-Coated Electrochromic Disubstituted 3,4-Propylenedioxythiophene Polymers. ACS Appl. Mater. Interfaces.

[20] Hizalan G, Balan A, Baran D, Toppare L (2011). Spray Processable Ambipolar Benzotriazole Bearing Electrochromic Polymers with Multi-colored and Transmissive States. J. Mater. Chem..

[21] Reeves BD (2004). Spray Coatable Electrochromic Dioxythiophene Polymers with High Coloration Efficiencies. Macromolecules.

[22] Vasilyeva SV (2011). Material Strategies for Black-to-Transmissive Window-Type Polymer Electrochromic Devices. ACS Appl. Mater. Interfaces.

[23] Beaujuge PM, Amb CM, Reynolds JR (2010). A Side-Chain Defunctionalization Approach Yields a Polymer Electrochrome Spray-Processable from Water. Adv. Mater..

[24] Beaujuge PM (2012). Structure-Performance Correlations in Spray-Processable Green Dioxythiophene-Benzothiadiazole Donor-Acceptor Polymer Electrochromes. Chem. Mater..

[25] Beaujuge PM, Ellinger S, Reynolds JR (2008). The Donor-Acceptor Approach Allows a Black-to-transmissive Switching Polymeric Electrochrome. Nat. Mater..

[26] Mi S (2015). AIEE-Active and Electrochromic Bifunctional Polymer and a Device Composed thereof Synchronously Achieve Electrochemical Fluorescence Switching and Electrochromic Switching. ACS Appl. Mater. Interfaces.

[27] Kerszulis JA, Bulloch RH, Teran NB, Wolfe RMW, Reynolds JR (2016). Relax: A Sterically Relaxed Donor-Acceptor Approach for Color Tuning in Broadly Absorbing, High Contrast Electrochromic Polymers. Macromolecules.

[28] Beaupre S, Breton AC, Dumas J, Leclerc M (2009). Multicolored Electrochromic Cells Based On Poly(2,7-Carbazole) Derivatives For Adaptive Camouflage. Chem. Mater..

[29] Sicard L, Navarathne D, Skalski T, Skene WG (2013). On-Substrate Preparation of an Electroactive Conjugated Polyazomethine from Solution-Processable Monomers and its Application in Electrochromic Devices. Adv. Funct. Mater..

[30] Li HZ (2016). Spray Coated Ultrathin Films from Aqueous Tungsten Molybdenum Oxide Nanoparticle Ink for High Contrast Electrochromic Applications. J. Mater. Chem. C.

[31] Li KR, Zhang QH, Wang HZ, Li YG (2016). Lightweight, Highly Bendable and Foldable Electrochromic Films Based on All-Solution-processed Bilayer Nanowire Networks. J. Mater. Chem. C.

[32] Romero R, Dalchiele EA, Martin F, Leinen D, Ramos-Barrado JR (2009). Electrochromic Behaviour of Nb2O5 Thin Films with Different Morphologies Obtained by Spray Pyrolysis. Solar Energy Mater. Solar Cells.

[33] Chang CW, Liou GS, Hsiao SH (2007). Highly Stable Anodic Green Electrochromic Aromatic Polyamides: Synthesis and Electrochromic Properties. J. Mater. Chem..

[34] Chuang YW, Yen HJ, Wu JH, Liou GS (2014). Colorless Triphenylamine-Based Aliphatic Thermoset Epoxy for Multicolored and Near-Infrared Electrochromic Applications. ACS Appl. Mater. Interfaces.

[35] Yen HJ, Liou GS (2009). Solution-Processable Novel Near-Infrared Electrochromic Aromatic Polyamides Based on Electroactive Tetraphenyl-p-Phenylenediamine Moieties. Chem. Mater..

[36] Chang CW, Chung CH, Liou GS (2008). Novel Anodic Polyelectrochromic Aromatic Polyamides Containing Pendent Dimethyltriphenylamine Moieties. Macromolecules.

[37] Liou GS, Chang CW (2008). Highly Stable Anodic Electrochromic Aromatic Polyamides Containing N,N,N’,N’-tetraphenyl-p-phenylenediamine Moieties: Synthesis, Electrochemical, and Electrochromic Properties. Macromolecules.

[38] Hsiao SH, Liou GS, Kung YC, Yen HJ (2008). High Contrast Ratio and Rapid Switching Electrochromic Polymeric Films Based on 4-(dimethylamino)triphenylamine-functionalized Aromatic Polyamides. Macromolecules.

[39] Liou GS, Hsiao SH, Huang NK, Yang YL (2006). Synthesis, Photophysical, and Electrochromic Characterization of Wholly Aromatic Polyamide Blue-Light-Emitting Materials. Macromolecules.

[40] Chang CW, Liou GS (2008). Novel Anodic Electrochromic Aromatic Polyamides with Multi-stage Oxidative Coloring Based on N, N, N’, N’-tetraphenyl-p-phenylenediamine Derivatives. J. Mater. Chem..

