# Supplementary material for: Controllable Electrochromic Polyamide Film and Device Produced by Facile Ultrasonic Spray-coating
Source: Sci Rep. 2017 Sep 20;7:11982. doi: 10.1038/s41598-017-11862-1 (PMC5607338; doi:10.1038/s41598-017-11862-1)
Supplement: Supplementary file 1 — Controllable Electrochromic Polyamide Film and Device Produced by Facile Ultrasonic Spray-coating [file 41598_2017_11862_MOESM1_ESM.pdf]

Supplementary Information

Controllable Electrochromic Polyamide Film and Device Produced by Facile Ultrasonic Spray-coating

Huan-Shen Liu[1,+], Wei-Chieh Chang[2,+], Chin-Yen Chou[1], Bo-Cheng Pan[1], Yi-Shan Chou[2], Guey-Sheng Liou[1,*], and Cheng-Liang Liu[2,*]

[1]Institute of Polymer Science and Engineering, National Taiwan University, Taipei, 10617 Taiwan

[2]Department of Chemical and Materials Engineering, National Central University, Taoyuan, 32001 Taiwan

*gsliou@ntu.edu.tw; clliu@ncu.edu.tw

[+]These authors contributed equally to this work

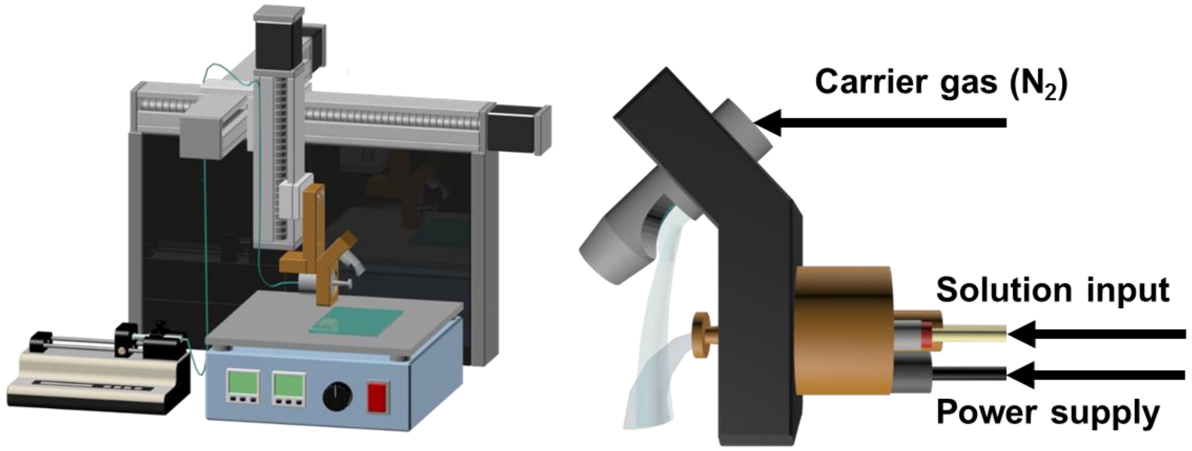

Figure S1. Schematic representation of setup for ultrasonic spray-coating of electrochromic polymer thin film.

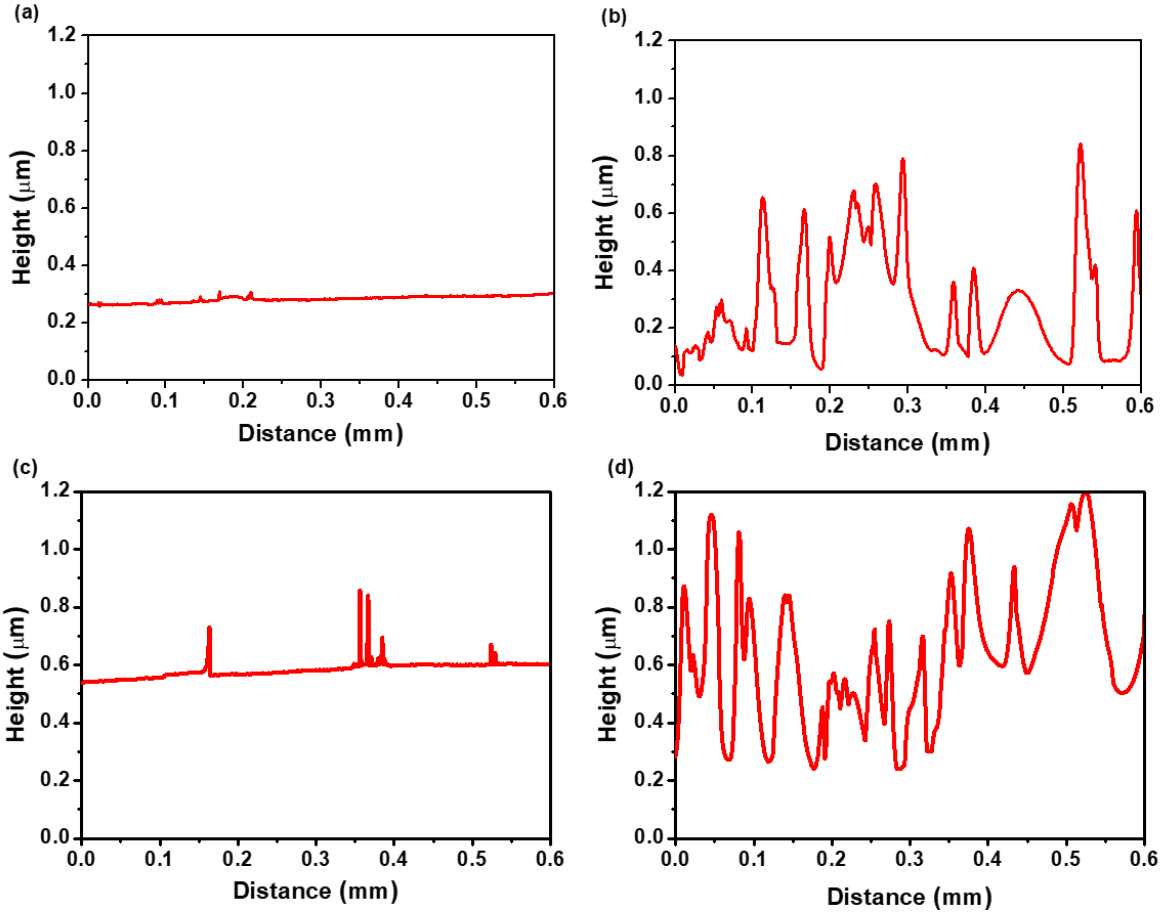

Figure S2. Surface height line scans of ultrasonic spray-coated (a) **Film A**, (b) **Film B**, (c) **Film C** and (d) **Film D**, measured by profilometry over a lateral distance of 0.6 mm.

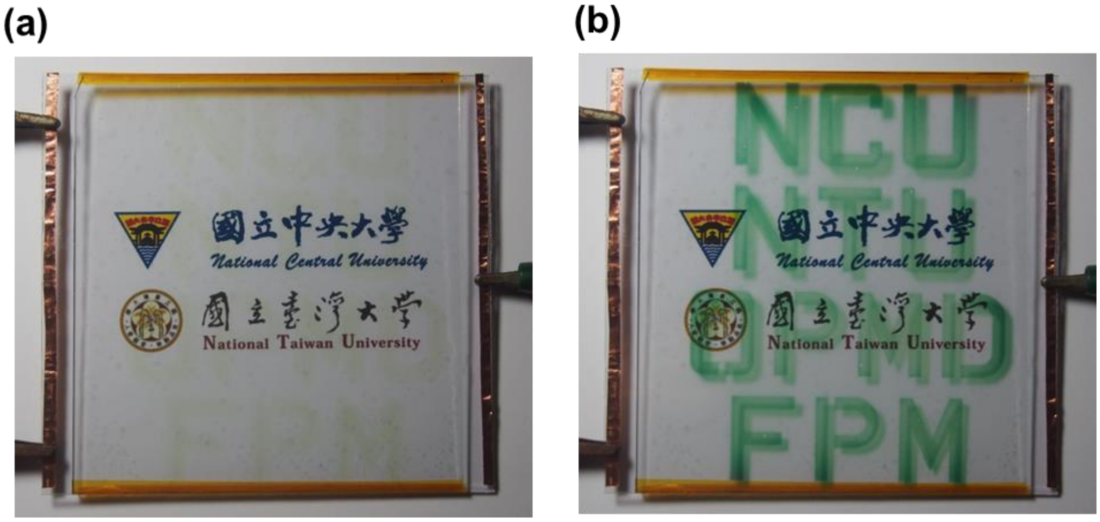

Figure S3. Photograph of the pattern TPA-OMD polyamide ECD in the (a) neutral state and (b) oxidized state.
